# How does Independent Component Analysis Preprocessing Affect EEG Microstates?

**DOI:** 10.1007/s10548-024-01098-4

**Published:** 2025-02-04

**Authors:** Fiorenzo Artoni, Christoph M. Michel

**Affiliations:** 1 Department of Clinical Neurosciences, Faculty of Medicine, Université de Genève, Geneva, Switzerland; 2 Department of Electronics, Information and Bioengineering, Politecnico di Milano, Milan, Italy; 3 Department of Basic Neurosciences, Faculty of Medicine, Université de Genève, Campus Biotech, Geneva, Switzerland; 4 CIBM Center for Biomedical Imaging, Lausanne, Geneva, Switzerland

**Keywords:** EEG, Microstates, Preprocessing, Independent component analysis, Artifact removal

## Abstract

Over recent years, electroencephalographic (EEG) microstates have been increasingly used to investigate, at a millisecond scale, the temporal dynamics of large-scale brain networks. By studying their topography and chronological sequence, microstates research has contributed to the understanding of the brain’s functional organization at rest and its alteration in neurological or mental disorders. Artifact removal strategies, which differ from study to study, may alter microstates topographies and features, possibly reducing the generalizability and comparability of results across research groups. The aim of this work was therefore to test the reliability of the microstate extraction process and the stability of microstate features against different strategies of EEG data preprocessing with Independent Component Analysis (ICA) to remove artifacts embedded in the data. A normative resting state EEG dataset was used where subjects alternate eyes-open (EO) and eyes-closed (EC) periods. Four strategies were tested: (i) avoiding ICA preprocessing altogether, (ii) removing ocular artifacts only, (iii) removing all reliably identified physiological/non physiological artifacts, (iv) retaining only reliably identified brain ICs. Results show that skipping the removal of ocular artifacts affects the stability of microstate evaluation criteria, microstate topographies and greatly reduces the statistical power of EO/EC microstate features comparisons, however differences are not as prominent with more aggressive preprocessing. Provided a good-quality dataset is recorded, and ocular artifacts are removed, microstates topographies and features can capture brain-related physiological data and are robust to artifacts, independently of the level of preprocessing, paving the way to automatized microstate extraction pipelines.

## Introduction

Over recent years electroencephalography (EEG) microstates research has grown exponentially in popularity given its potential to contribute to more sophisticated diagnosis, monitoring, prognosis and prevention of mental disorders in various fields, particularly clinical psychology and psychiatry (Kleinert et al. 2023). Microstates allow to investigate at a millisecond scale, the temporal dynamics of large-scale brain networks (Michel and Koenig 2018), which are characterized by a discrete number of patterns of synchrony across the cortex that remain stable for 40–120ms before rapidly transitioning to a different state (Lehmann et al. 1987). One way of computing microstate topographies is to perform k-means cluster analysis (Pascual-Marqui et al. 1995). The temporal dynamics of microstates can then be analyzed by extracting characteristics such as average duration, average number of occurrences of one particular microstate in a period of time, variance explained, and, more recently, microstates syntax and complexity (Artoni et al. 2023; Tait et al. 2020).

An analysis of published work shows remarkable similarities of microstates topographies across studies, which led to the identification of several “canonical” microstates typically observed in resting states EEG data (Koenig et al. 2023; Tarailis et al. 2023; Zanesco 2023). However, a closer analysis of published studies highlighted considerable topographic variance in the template maps assigned to the same microstate class (Michel and Koenig 2018). Such variability may depend on a variety of factors, including the type of algorithm used for microstates extraction or manual mis-classification of the class microstates belong to (Koenig et al. 2023). Another possibly relevant source of variability across studies may be the type of pipelines and artifact removal techniques performed to preprocess the data before microstates extraction. Currently, as long as microstates do resemble those found in literature in comparable studies, data are considered by authors and readers to have been preprocessed well. However, different artifact removal strategies may alter microstate topographies and resulting features.

Independent Component Analysis (ICA) is one of the most popular algorithms for removal of artifacts from EEG. ICA, in fact, allows to disentangle information linearly mixed at the scalp EEG electrodes into maximally temporally independent component (IC) processes that can be used to assess individual EEG effective source dynamics without the requirement of the definition of an explicit electrical forward problem head model (Makeig et al. 1996, 2004). Each IC is represented by a pattern of relative projection to the scalp channel (often referred to as “scalp map”) and by the time varying signed equivalent source signal, often referred to as IC time course (Delorme et al. 2012). Provided enough adequately recorded and preprocessed data are available, ICA has been found to well separate from brain data classes of stereotyped artifacts such electrocardiographic (ECG) signal contamination, scalp and neck muscles electromyographic (EMG) activities, electro-oculographic (EOG) activity as a result of lateral eye movements, eye blinks and ocular motor tremor and single-channel noise, produced by occasional disruption of the connection between scalp and electrodes (Makeig et al. 2004; Onton et al. 2006).

Using ICA artifacts removal as a microstate preprocessing step requires obtaining highly reliable extracted components and their correct interpretation and use in further analysis. Sometimes, however, noise in the data (originating from inadequate data acquisition, low-impedance scalp/sensor interfaces small, irresolvable signal sources), inadequate data sampling (e.g., not enough data points are available) and algorithmic shortcomings (e.g., convergence issues, presence of local minima) may reduce the quality of ICs and make it difficult to reliably identify any but the ICs with the highest explained variance (i.e., ocular activity)(Artoni et al. 2014; Delorme et al. 2007; Jung et al. 2000). A possible approach to preprocessing is that of performing a first, more aggressive filtering (e.g., with higher high-pass frequency to increase data stationarity), extract ICA components, and use them as spatial filters after a more conservative preprocessing procedure (e.g. lower high-pass frequency). This is often an advantage for example when low frequencies (< 1 Hz) should not be removed, e.g., in experiments involving sleep/unconsciousness (Artoni et al. 2022).

To help identify which ICs to retain or discard from further analysis, several measures have been described in literature, for example by evaluating the stability to data resampling (Artoni et al. 2014; Groppe et al. 2009), mutual information reduction (Palmer et al. 2012) or the dipolarity of IC topographies (i.e., the goodness of fit of a single equivalent dipole to the IC scalp topography)(Delorme et al. 2012). Sorting of ICs into classes is further aided by automatic classification methods such as ICLabel (Pion-Tonachini et al. 2019). Even after correct ICs identification, however, there is currently no definite set of rules as to which IC removal strategy is best suited for microstates extraction.

Broadly speaking, it is possible to identify at least four IC removal strategies in decreasing levels of conservativeness, i.e., (i) avoiding ICA preprocessing altogether (i.e., using raw data, after band-pass filtering, bad channel interpolation, and bad epochs rejection), (ii) removing ocular ICs only (blinks and eye movements), (iii) removing all ICs that could be reliably identified as physiological artifacts (e.g. heartbeat, muscles), (iv) removing all ICs except those that can be reliably identified as brain ICs based on ICLabel output probabilities and dipolarity (Delorme et al. 2012) – see Methods. The aim of this work is to test the reliability of the microstate extraction process, i.e., the stability of microstate features against these four strategies of ICA-based EEG data preprocessing with a normative resting state EEG dataset where subjects alternate eyes-open and eyes-closed periods.

## Materials and Methods

### Experimental Setup and Data Collection

The data were collected between 2013 and 2015 from 227 participants, recruited as part of the Leipzig Study for Mind–Body–Emotion Interaction study of the Max Plank Institute. The dataset comprised group samples of younger (*N* = 153, 45 females, mean age 25.1 years, SD = 3.1) and older (*N* = 74, 37 females, mean age 67.6 years, SD = 4.7) adults who underwent an extensive medical and psychological screen procedure prior to study inclusion, and tested at the Day Clinic for Cognitive Neurology of the University Clinic and the Max Plank Institute for Human and Cognitive and Brain Sciences in Leipzig, Germany. All participants provided written informed consent prior to study participation, received monetary compensation for the time invested in the study, and agreed to anonymous data sharing. Data were collected and shared by (Babayan et al. 2019) in accordance with the Declaration of Helsinki and the study protocol was approved by the ethics committee of the University of Leipzig (reference #154/13-ff). Further details regarding participants recruitment and eligibility are reported by (Babayan et al. 2019).

Resting state EEG was recorded from 216 participants in a quiet chamber. Subjects sat in front of a screen display and alternated eyes closed and eyes open conditions for a total of 16 contiguous 1 min blocks. In eyes-closed conditions subjects were asked to remain awake and, in eyes-open conditions, to sit still while fixating a black cross presented on a white background. Notifications of the changes between blocks were given participants by means of the Presentation software (Neurobehavioral Systems Inc.) and marked within the data.

The EEG data was recorded via a 62-channel active electrodes cap (ActiCAP, Brain Products, GmbH, Germany), arranged according to the international 10–10 system for electrodes positioning (Oostenveld and Praamstra 2001), with an additional electrode below the right eye to capture vertical eye movements (vEOG). The reference was set at position FCz, the ground was located at the sternum. Data were acquired with a BrainAmp MR plus amplifier (Brain Products GmbH, Germany) with a sampling rate of 2500 Hz, 0.1 µV as amplitude resolution, and hardware filtered between 0.015 Hz and 1 kHz.

### Data Preprocessing

**Fig. 1:**
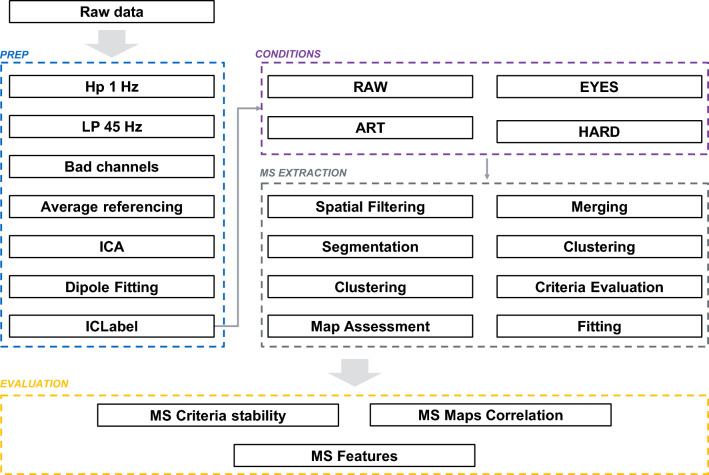
Schematics representing the steps performed for the evaluation of the influence of preprocessing on Microstates. Data preprocessing steps (blue dashed box) include a preliminary high-pass filtering of the data (1Hz), low pass filtering (45Hz), removal of channels and extraction of Independent Components - ICs (algorithm: AMICA). A best-equivalent dipole is then fitted to each IC scalp map. ICs are automatically labelled by means of ICLabel and sorted according to dipolarity (i.e., complement to 1 of the residual variance – Rv – of the corresponding equivalent dipole). ICs are then removed from the data, according to four different levels. In condition “RAW”, no ICs are removed; in condition “EYES” only vertical and lateral ocular artifact ICs with high dipolarity (> 85% - see methods) are removed; in condition “ART” all IC with high dipolarity and categorized as physiological artifacts with high confidence (ICLabel probability > 80%), such as eye activity, muscular activity, heart are removed. In “HARD” conditions all ICs are removed except for those classified as brain activity with ICLabel probability > 80% and with high dipolarity (> 85%). Remaining data for each condition then is sent to a microstate extraction pipeline (grey dashed box) comprising a 1^st^ level clustering (spatial filtering, segmentation, GFP clustering, Map assessment) and a 2^nd^ level clustering (merging, 1^st^ level maps clustering, evaluation of criteria to determine the best number of microstates and fitting). Microstates for each preprocessing condition are finally evaluated according to criteria stability, correlation across maps and classic Microstate features (see Methods)

A diagram of the full data processing pipeline is shown in Fig. 1. Raw data were first high-pass filtered using a zero-phase 1 Hz 24th order Chebyshev type II filter, low-pass filtered using a zero-phase 45 Hz, 70th order Chebyshev type II filter, and finally resampled at 250 Hz. Thanks to the rollover steepness of the filter used, no further 50 Hz comb Notch Filters were necessary. Artifact channels with extreme amplitude artifacts, i.e., with Kurtosis and/or amplitude range outside a threshold of 6 standard deviations with respect to other channels were removed. Remaining data were average re-referenced and single-model independent components (ICs) were extracted via the AMICA algorithm (Palmer et al. 2012), after reducing by one dimensionality by means of principal component analysis (PCA) to account for average reference. Among ICA algorithms, AMICA yields the best mutual information reduction (Delorme et al. 2012). To increase ICs stability, automatic rejection of data with log-likelihood outside the range of 5 standard deviations, was performed 3 times, after the first 5 iterations at 5 steps intervals (Artoni et al. 2017; Klug et al. 2022). The maximum number of iterations was set to 2000. Note that data rejection was an internal step within the AMICA algorithm to increase the reliability of extracted ICs. IC weights were then applied to the pre-ICA dataset (i.e., without data rejection).

Dipolar source localization of resulting IC scalp topographies (columns of the mixing matrix) was performed via the DIPFIT plugin (Delorme et al. 2012), within EEGLAB (Delorme and Makeig 2004). The dipolarity of each IC was defined as $\:dip\left({IC}_{n}\right)\:=\:100(1-resvar({IC}_{n}\left)\right)$ where $\:resvar\left({IC}_{n}\right)$ is the residual variance (fitting error) between its scalp map projection and that of its single equivalent dipole (or double, in case of an ocular IC), here computed using a best-fitting spherical four shell model of the head (radius: 71, 72, 79, 85 mm, shell conductance: 0.33, 0.0042, 1, 0.33 µS). Dipolar ICs have dipolarity values over 85%, and quasi-dipolar ICs have dipolarity values above 95% (Artoni et al. 2014; Delorme et al. 2012).

ICLabel (Pion-Tonachini et al. 2019) was then used to assign each IC a probability of belonging to different categories, namely:

- Brain, i.e., ICs containing activity generated by locally synchronous activity in one or two well-connected cortical patches, often with decreasing power at higher frequencies.
- Muscle, i.e., ICs with activity originating from groups of muscle motor units, usually with high-frequency broadband activity aggregating several motor unit action potentials.
- Eye, i.e., ICs with quick or sustained “square” DC-shifts originating from eye blinks and lateral eye movements respectively. Rotation of the eyes shifts the projection of the electrical dipoles they form (negative pole at the retina, positive at the cornea) to the frontal scalp (Malmivuo and Plonsey 1995).
- Heart, i.e., ICs that exhibit a clear QRS complex in their time series and often result in topographical scalp maps approximating a diagonal linear gradient.
- Line noise, i.e., ICs that capture the effect of stationary line current noise, with high power peaks at 50 Hz and 60 Hz, often originating from electrical fixtures or improperly grounded EEG amplifiers.
- Channel noise, i.e., ICs that indicate the presence of large artifacts affecting single channels or poor signal quality due e.g., to high impedance physical electrode movement.
- Others, i.e., ICs that do not fit into any of the other types for example containing indeterminate noise or that were not well separated by the ICA algorithm.

**Fig. 2:**
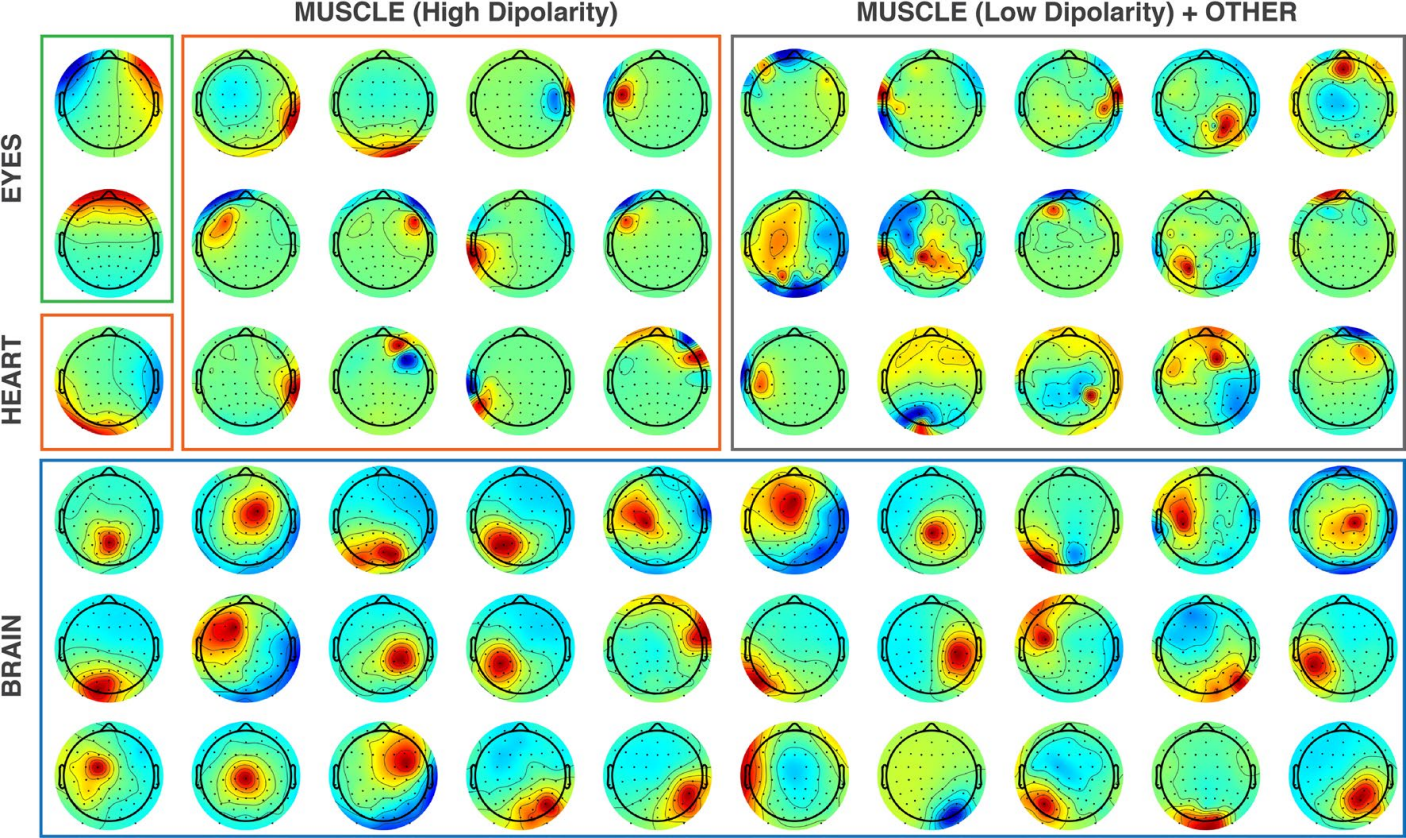
Labeling of ICs. Independent component (IC) sorting process for one exemplar subject. In RAW condition all ICs are retained. In EYES condition, ocular artifact ICs (green box), with high dipolarity (> 85%) and high ICLabel probability (> 0.8) are removed. In ART condition all artifact ICs with high dipolarity (EYES, HEART, MUSCLE) and high ICLabel probability are removed. In HARD condition all ICs are removed except for highly dipolar brain activity ICs identified as such by ICLabel with high probability. Note that muscle artifact ICs and other artifacts, even if identified as such by ICLabel with high probability, that are not highly dipolar (and thus possibly a result of improper ICA convergence) are only removed in HARD condition

To compare the influence of different IC removal strategies on microstates parameters, data were processed according to 4 levels of preprocessing, i.e., “cleaning intensities”, based on the types and number of ICs removed. Figure 2 shows a representation of the sorting process for one exemplar subject. Removal of ICs is performed by back-projecting the IC activation data after zeroing out the columns of the mixing matrix corresponding to the artifact ICs.

- In RAW preprocessing condition, no ICs are removed from the data.
- In EYES condition, only ICs classified as ocular artifacts with high ICLabel probability ($\:{p}_{ICLabel}$> 0.80) and dipolarity $\:dip\left({IC}_{n}\right)$> 0.85 are removed (green box).
- In ART condition, all physiological artifact ICs that could be reliably classified as such are removed. This type of preprocessing corresponds to a conservative approach that limits the removal of possibly physiological data. Components removed are those classified as ocular artifacts (as in EYES condition), heart activity ($\:{p}_{ICLabel}$> 0.80), and highly dipolar muscular activity ($\:{p}_{ICLabel}$> 0.80, $\:dip\left({IC}_{n}\right)$> 0.85). Low-dipolarity or low-probability muscle activity might in fact be due to non-stationary noise, active at certain periods of time or to improperly separated ICs
- The HARD condition corresponds to the least conservative preprocessing, where all ICs that were not reliably classified as brain activity (i.e., $\:{p}_{ICLabel}$> 0.80, $\:dip\left({IC}_{n}\right)$> 0.80) are removed. This process ensures the cleanest resulting signal, at the cost of possibly discarding a certain amount of physiological data.

The dipolarity threshold was assigned based on the fact that dipolar ICs with dipolarity greater than 80/85% have a high probability to be also stable to point-by-point bootstrapping (Artoni et al. 2014; Delorme et al. 2012). Regarding ICLabel, a preliminary analysis revealed that ICs, when correctly assigned (based on expert review) were given a probability of at least 0.85/0.90 to the correct class.

### Extraction of Microstates

Microstates extraction was performed within Cartool (Brunet et al. 2011) in two stages. The first stage of microstates extraction was performed independently for each preprocessing level (RAW, EYES, ART, HARD) and condition (eyes open - EO, eyes closed - EC). EEG microstate segmentation steps are outlined in Fig. 1 and also described in (Murray et al. 2008).

For each dataset, missing channels were first interpolated using a spherical spline interpolation within Cartool (Michel and Brunet 2019). Then the Global Field Power (GFP) maxima were extracted. For each preprocessing level, condition and participant, the GFP peak topographies (channel values at the timestamp corresponding to the GFP peak) were extracted and clustered via modified k-means to extract distinct templates (Koenig et al. 2002; Murray et al. 2008; Pascual-Marqui et al. 1995). The spatial correlation between each GFP map and each randomly generated template was calculated while ignoring the polarity of maps (Michel and Koenig 2018).

**Fig. 3:**
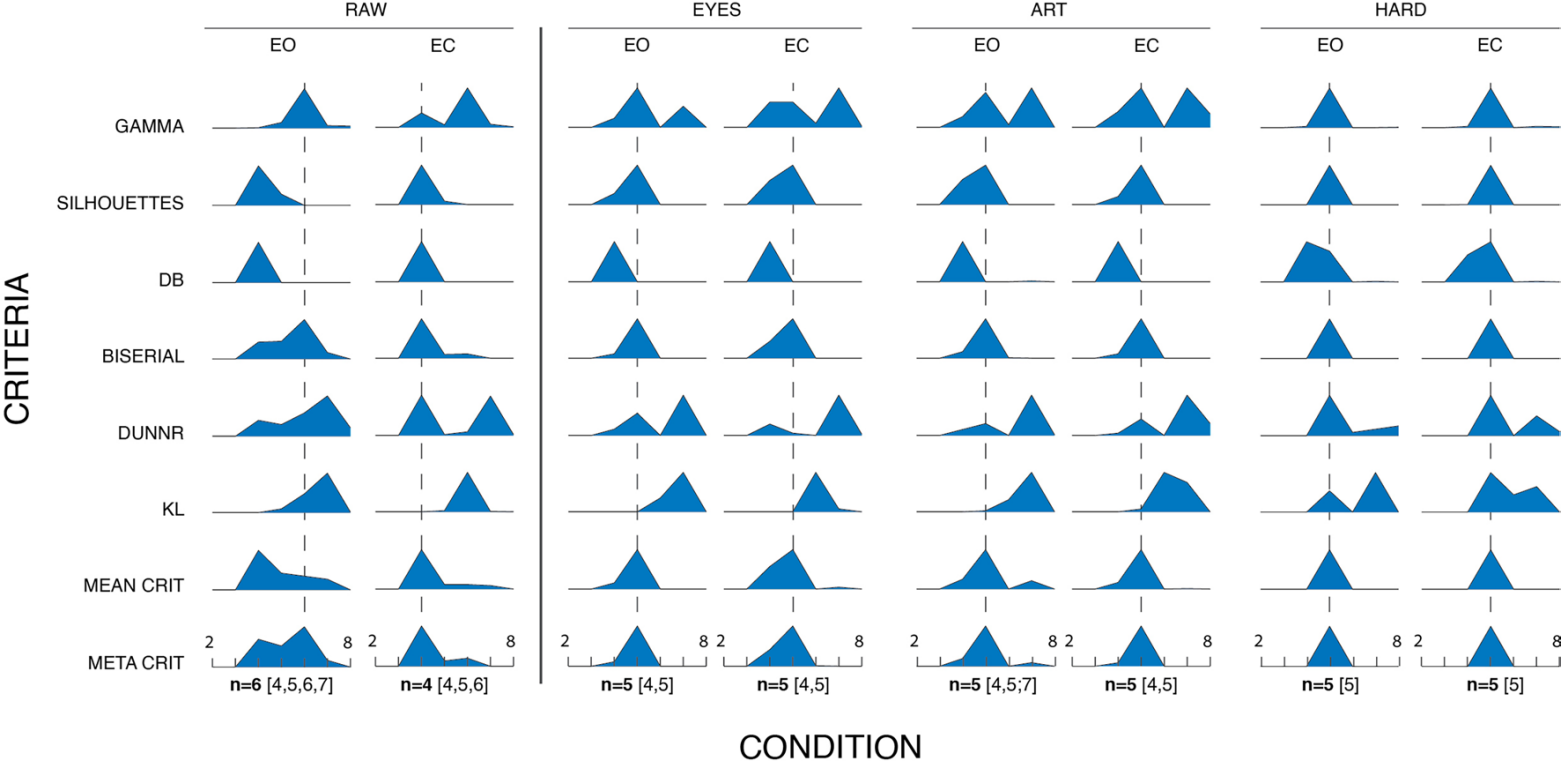
Microstates extraction assessment. Stability assessment of criteria to determine the best number of microstates to retain in further analyses for each preprocessing condition. The criteria used were “Gamma” (GAMMA), Point-Biserial (BISERIAL), Davies-Bouldin (DB), Dunn Robust (DUNNR), “Krzanowski - Lai” (KL), “Silhouettes (SILHOUETTES)”. These criteria were also combined into a “Mean criterion” (MEAN CRIT), that is a criterion representing the “average” of the probabilities yielded by all the other criteria and the “Meta criterion” (META CRIT) that represents the best principled choice for the number of microstates. For each physiological resting state condition analyzed (eyes open – EO, eyes closed – EC) and assessment criteria (“GAMMA” through “META CRIT”) a plot shows the probability (according to the specific criterion) for each number of microstates (2 to 8). A dashed vertical line for each column represents the best number of microstates according to the metacriterion for the relative condition (also highlighted in bold at the bottom). In RAW condition EO and EC do not converge on the same optimal number of microstates n=6 and n=4 respectively). Possible microstate solutions highlighted by the meta criterion also range from 4 to 7 microstates. The greater the removal of ICs, the greater the meta criterion variability (EYES: 4,5; ART: 4,5,7; HARD: 5). Except for RAW condition, removal of artifact ICs (even just ocular artifacts) ensures convergence at n = 5 microstates for both eyes open and eyes closed physiological conditions.

Each template was then iteratively updated by averaging the GFP maps that presented the highest correlation with the template. At the same time, the Global Explained Variance (GEV) of template maps was calculated, and the process was iterated until the stability of GEV was reached. For each condition (EO and EC) and preprocessing level (RAW, EYES, ART, HARD) the optimal number of microstate classes was determined using different criteria (Fig. 3), each estimating the “quality” of a single segmentation according to specific metrics (Charrad et al. 2014; Krzanowski and Lai 1988; Milligan and Cooper 1985). Criteria results were merged to define respectively a mean/meta-criterion as the mean/median of all optimal numbers of clusters across all criteria (Bréchet et al. 2019; Custo et al. 2017). The criteria used were:

- Gamma: Goodman and Kruskal adaptation based on concordant vs. discordant clustered pairs.
- Silhouettes: Goodness of fit evaluation of each cluster consistency.
- Davies and Bouldin: A measure derived from the ratio of within-cluster and between-cluster separation.
- Point-Biserial: A measure of correlation calculated between a binary cluster index and distance matrix.
- Dunn: An evaluation of the degree of separation between all clusters.
- Krzanowski-Lai Index: A measure of within-clusters dispersion.

The dominant microstates were identified within each condition from the templates across participants using a second modified k-means clustering step. Each clustering step was computed 200 times to maximize stability and to overcome the possible statistical instability of the randomization procedure within the k-means algorithm (Murray et al. 2008). The spatial correlation was finally computed between microstates across all conditions

### Fitting and Microstate Features

**Fig. 4:**
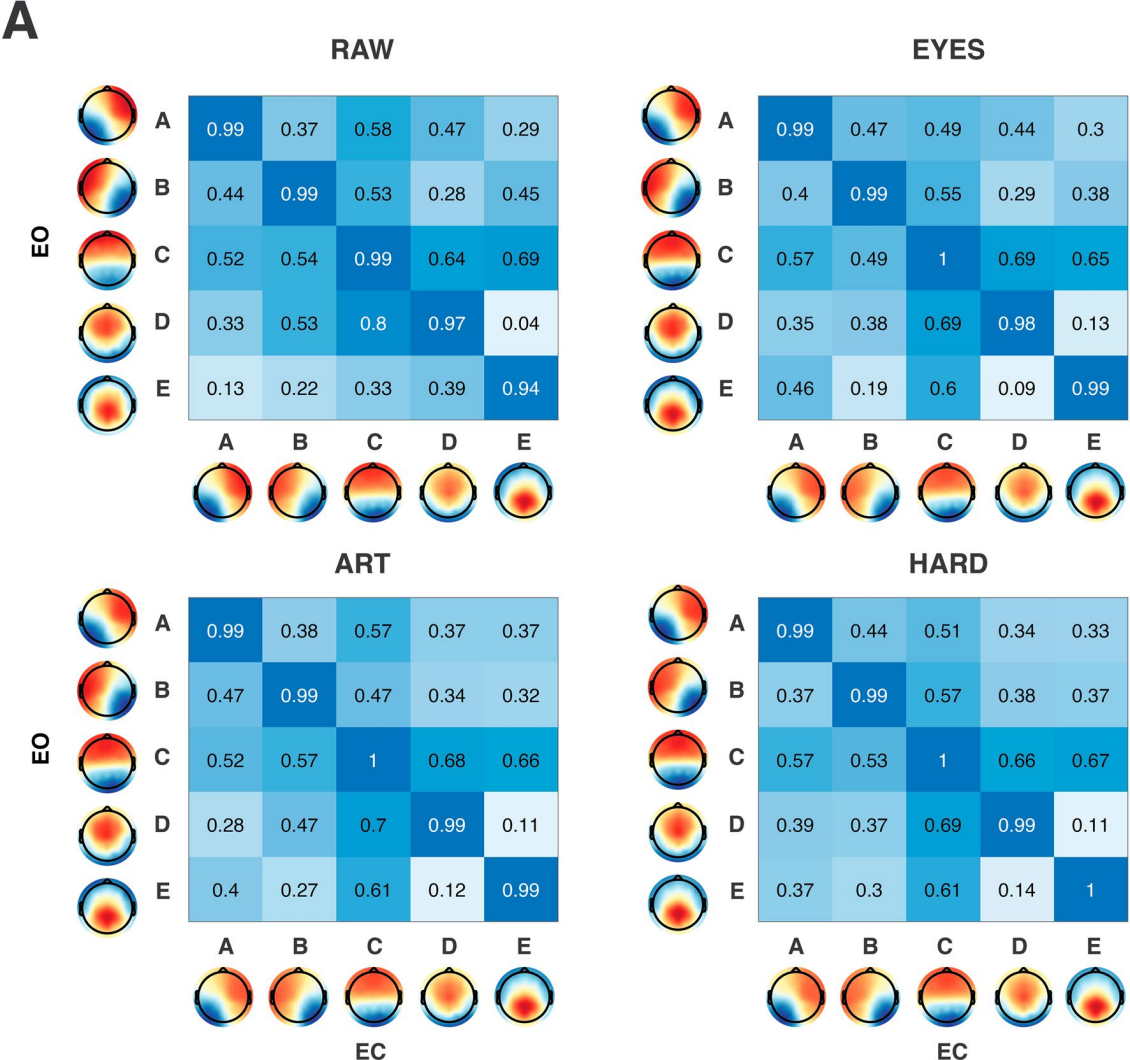
Microstate Scalp Topographies. Cross-correlation of paired eyes-open (EO) and eyes-closed (EC) scalp topographies for each preprocessing condition (RAW, EYES, ART, HARD)

**Fig. 5:**
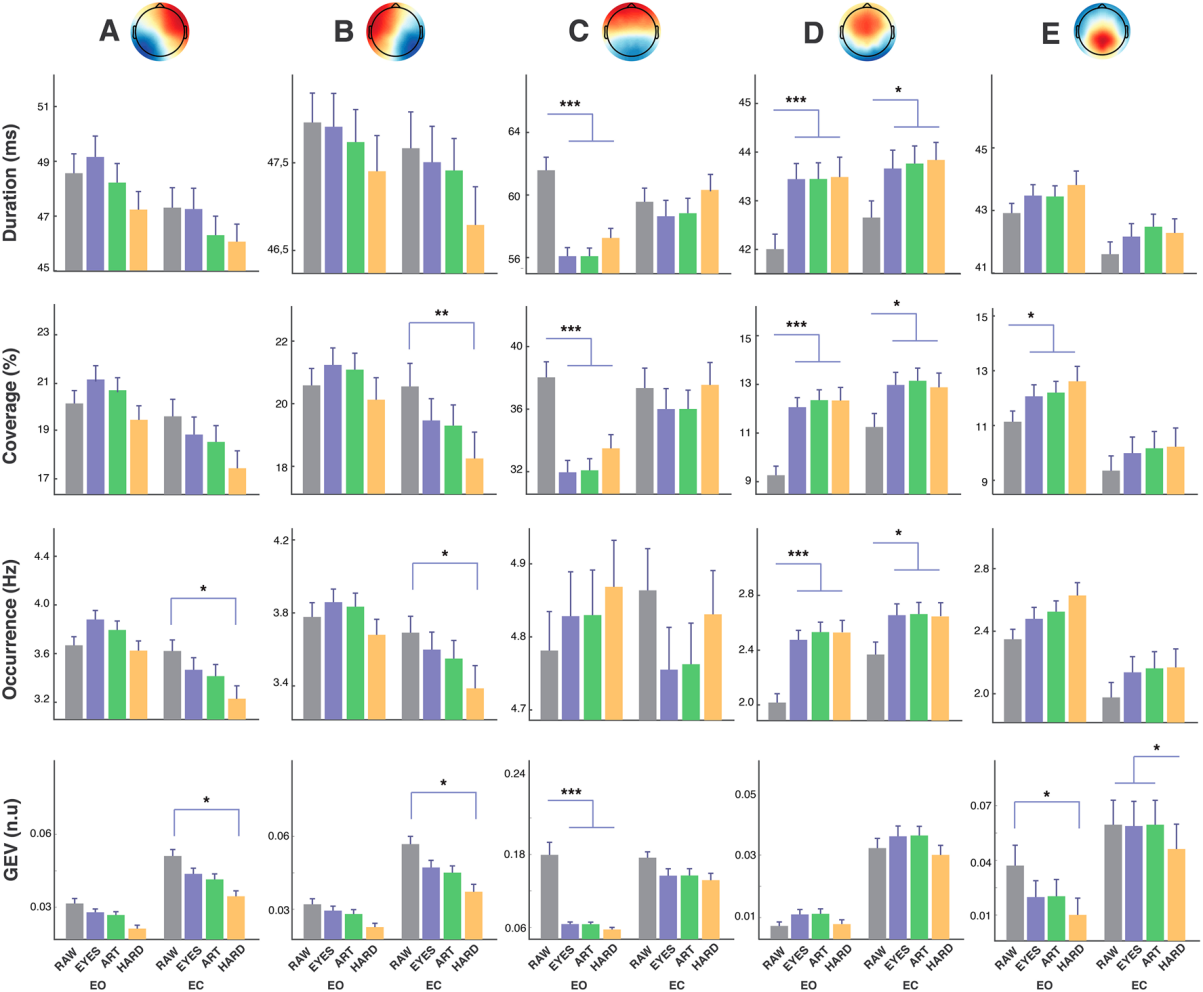
Evaluation of microstates results. Comparison of duration, coverage, and occurrence and Global Explained variance values between physiological conditions (eyes open – EO and eyes closed – EC), for the 4 different preprocessing conditions (RAW, EYES, ART, HARD). To preserve readability statistical significance is displayed within preprocessing conditions. * p<0.05, ** p<0.01, ***  p<0.001

The topographical correlation values between EO and EC condition for each microstate and preprocessing level is reported in Fig. 4. Given the high correlation between paired maps across conditions, for each preprocessing level, the data of both EO and EC conditions were pooled and the extraction of microstates repeated. As standard procedure for fitting of microstates, the spatial correlation between the templates (i.e., microstates topographies A-E) identified at the group level and each EEG frame of each original EEG dataset was computed using a temporal constraint (Segments Temporal Smoothing) of 6 samples (24 ms) (Artoni et al. 2022). Each EEG frame was then labeled in a “winner-takes-all” manner (Michel and Koenig 2018) according to the group template (i.e., microstate) it best corresponded to, which generated the microstate sequence for further analysis. No labeling was performed at correlations lower than 0.5. Microstate features of each subject for each condition (EO, EC), preprocessing level (RAW, EYES, ART, HARD) were compared via a 3-way ANOVA (condition x preprocessing level x map), with Tukey’s adjustment for multiple comparisons and reported in Fig. 5. Features compared were:

- *Duration*, obtained for each microstate by averaging the time said microstate is active (in a winner-takes-all fashion) before transitioning to another microstate.
- *Occurrence*, computed for each microstate as the number of occurrences of said microstate per second of data.
- *Coverage*, computed for each microstate as the relative number of time points of the dataset covered by said microstate.
- *Global Explained Variance (GEV)*, i.e., the percentage of data variance explained by a given set of microstate maps.

## Results 

###  Labeling of ICs

On average, a total of 2 ± 1 (Median ± Median Absolute Deviation) ICs were reliably identified as ocular artifacts (EYES - green box Fig. 2). An average of 4 ± 1 ICs were reliably identified as belonging to condition ART (green and orange boxes). Finally an average of (34 ± 4) ICs were removed in the HARD condition (green, orange and gray contours). The percent of variance accounted for by removed ICs in EO condition was generally higher compared to EC (Table 1).

**Table 1:** Percent of variance accounted for (Mean ± Standard deviation) by the independent components removed in EYE, ART and HARD preprocessing levels and eyes open (EO)/eyes closed (EC) conditions

	EYE	ART	HARD
EO	(27.9 ±15.7)	(29.6 ± 16.4)	(47.6 ± 20.6)
EC	(10.6 ± 8.5)	(12.1 ± 9.4)	(29.3 ± 17.9)

### Microstates Assessment

In RAW condition, microstates assessment criteria reveal a different optimal number of microstates for eyes open (*n* = 6) and eyes closed (*n* = 4) conditions respectively (Fig. 3). This number stabilizes to *n* = 5 after any degree of IC removal. Increasing the preprocessing level also reduced the range of possible numbers of microstates yielded by the meta-criterion (RAW: EO = 4,5,6,7; EC = 4,5,6. EYES: EO = 4,5; EC = 4,5. ART: EO = 4,5,7; EC = 4,5. HARD: EO = 5; EC = 5).

Figure 4 shows high topographical scalp map correlations for each microstate between EO and EC, almost identical across EYES, ART, HARD preprocessing levels and slightly lower for RAW, especially for microstates D (correlation 0.97) and E (correlation 0.94).

### Comparison of Features

Figure 5 represents the final topographical microstate maps and fitting results. To avoid the risks of overfitting outlined in (Murphy et al. 2023), a single microstates set, computed by taking into account all conditions and preprocessing levels, was used for fitting.

Overall, statistical analyses reveal significant interaction (*p* < 0.001) with “map”, “preprocessing” and “condition”, for all features. In addition, occurrence and GEV are significatively different regarding preprocessing, condition and preprocessing/condition interaction. Duration differences are significant only considering condition and preprocessing/condition interaction.

More in details, regarding MS A, Duration, Coverage, Occurrence are significantly higher in EO compared to EC (*p* < 0.05) for all preprocessing levels except for RAW. GEV is significantly higher for EC compared to EO, regardless of preprocessing condition (*p* < 0.001). Considering preprocessing levels, significance is reached only between RAW and HARD in condition EC.

Similar considerations are true for MS B. However, for Duration, EO and EC differences are not significant in any preprocessing condition. Coverage, Occurrence and GEV are significantly higher for RAW compared to HARD in condition EC (*p* < 0.01).

While for MS A and B Duration and Coverage were higher for EO compared to EC, MS C duration and coverage show an inverse behavior with lower values for EO with respect to EC (*p* < 0.001). However, these differences are not significant for RAW (higher values for EO with respect to EC, though not significant). In EO condition, Duration and Coverage are significantly higher (*p* < 0.001) for RAW compared to other preprocessing levels. Single EO/EC comparisons of Duration, Coverage and GEV are all significant considering all preprocessing levels except for RAW.

Contrary to other microstates, MS D coverage and occurrence and duration in both EO and EC are significantly lower for RAW with respect to EYES, ART and HARD, although differences are more accentuated for EO (*p* < 0.001) than EC (*p* < 0.05). While GEV is lower in EO with respect to EC for all preprocessing conditions (*p* < 0.001), no significant differences could be observed across preprocessing levels. EO/EC comparisons do not reach significance, except for RAW, in Coverage and GEV.

Finally, MS E exhibits significant differences in duration, coverage, occurrence and GEV for EO with respect to EC. On the other hand, preprocessing level differences are significant for Coverage in EO (RAW vs. all other preprocessing levels, *p* < 0.05), for GEV in EO (RAW vs. HARD, *p* < 0.05) and GEV in EC (HARD vs. all other preprocessing levels, *p* < 0.05).

## Discussion

The results show a general stability of microstate features independently of the preprocessing level. Figure 5 shows that except for RAW, where ICA preprocessing is skipped altogether, differences of duration, coverage, occurrence and GEV across preprocessing levels (EYES, ART and HARD), do not reach significance regardless of condition (EO and EC). Relative differences across conditions (EO and EC) are not significantly altered by the level of preprocessing, except in case it is avoided altogether. Comparing EO and EC, MS A Duration, Coverage and Occurrence are significantly different only in the case of EYES, ART, HARD. Similarly, MS C Duration, Coverage and GEV, are significantly higher in EC compared to EO, for all preprocessing levels except for RAW where the values are similar.

Interestingly, removing ocular activity when extracting microstates does not adversely alter eyes-open/eyes-closed comparisons, at least considering microstate features. On the contrary, avoiding preprocessing increases variability in the data, so much so that EO/EC differences are mostly lost (EO/EC Duration, Coverage and Occurrence for MS A, B,C are similar). The only exception might be MS D, where preprocessing increases EO/EC similarity for Duration, Coverage and Occurrence. Furthermore, the metacriteria used to assess the optimal number of microstates for RAW, does not converge to *n* = 5, but rather to *n* = 6 and *n* = 4 respectively for EO and EC (Fig. 3). While this was not directly tested here, it is possible that a greater number of microstates is needed to explain ocular activity variance in EO condition, which is not the case for EC (*n* = 4). Interestingly, in all preprocessing levels (EYES, ART and HARD), the meta criterion converges to *n* = 5 regardless of condition, a result closest to the most accepted normative literature (Michel and Koenig 2018), which suggests a “homogenization” effect of artifact removal (at least ocular activity) through ICA, which seems to be beneficial to the process of microstates extraction. The effect is particularly evident when observing microstate topographies (Fig. 4): except for RAW MS E, where topographic correlation is lowest (*r* = 0.94), topographies are almost identical across conditions, with correlations very close to 1.

The greatest feature differences in microstates are observed when comparing any level of preprocessing (EYE, ART, HARD) with respect to performing no processing at all (RAW). Indeed the microstate extraction process relies on the clustering of repetitive quasi-stable topographies across time: the higher topographical variability, lower rate of occurrence and non-stationarity of most physiological and non-physiological artifacts (such as line noise, electrode displacements, movement artifacts, etc.) likely ensures their exclusion from 2nd level (and perhaps 1st level) clustering (see microstates extraction process described in the methods section), which is not be the case for ocular artifacts. Ocular artifacts have a very precise and stationary generator as they are produced by the rotation of the eye balls, movements of the eye lids and contraction of the extraocular muscles, highly variable across subjects, with magnitudes often 10 times larger (or more) than scalp EEG data, occurring often and with quite stable scalp topographies (with maximum amplitude at frontal EEG electrodes) (Croft and Barry 2000; Dimigen 2020; Hagemann and Naumann 2001). The scalp topography of eye blinks resembles the topography of microstate C. By not excluding eye blinks in the RAW condition, they will probably be labeled as microstate C in the winner-takes-all fitting step. This explains why in RAW, duration and coverage of Microstate C are comparable (if not higher) in the EO condition, while the opposite is true when eye blinks are removed by the ICA. Other artifacts do not produce scalp topographies that resemble the canonical microstates. The fitting procedure with the threshold of > 0.5 correlation will thus not label them with any of the microstates. In this sense, clustering and fitting “naturally” remove these artifacts, making more elaborated ICA artifact removal procedures unnecessary to obtain reliable microstates. However, it may also be interesting in future works to explore these conclusions with different fitting thresholds and/or non-competitive fitting.

The results also highlight that microstate topographies are stable regardless of preprocessing level, with the HARD level of preprocessing resulting in the highest data homogenization with metacriterion convergence to exactly *n* = 5 microstates **(**Fig. 3**)**. EO/EC differences are also preserved regardless of preprocessing (EYES, ART, HARD). However, different preprocessing strategies alter absolute values of microstate features (e.g., higher MS-A Duration/Coverage/Occurrence in EO for EYES compared to ART and HARD), although not significantly. This result further underlines the importance of carefully documenting preprocessing steps when comparing absolute values of microstate features across studies. Here convergence was reached for the extraction of 5 microstates: it is possible that the forced extraction of a larger number of microstates will also capture (and will be sensitive to) non-physiological or non-stationary artifacts. In that case, the differences between preprocessing levels might be more pronounced. Also, extracting a number of microstates outside the range suggested by the evaluation criteria (shown in Fig. 3) may reduce the reliability of the microstate extraction process and the comparison across works in literature.

These results have important implications as it becomes apparent that, for good quality resting state data, a simple ICA-based ocular artifact is sufficient to obtain reliable microstates. Such a pipeline, (e.g., by using ICLabel) can be easily automatized given the very distinct and recognizable IC scalp topographies and IC time courses of lateral and vertical ocular eye movements, further paving the way to the full automatization of the microstate extraction process.

It is important to note that the results presented have been extracted from good quality resting state data. Extreme amplitude artifact epochs should be rejected beforehand, and, if bad channels need to be interpolated, ICA should be computed after proper preliminary rank reduction (Artoni et al. 2018). Also, in case strong artifact contamination, it is possible to use a more aggressive preprocessing (ART/HARD) or else exclude such data from further analysis altogether. Indeed, it is always important to carefully consider whether to remove certain types of artifacts from the data. For example, ocular activity might be of interest in experiments involving visual tasks. In that case, ICA might be used to spatially filter the data and isolate ocular activity from other processes.

In conclusion, provided a good-quality dataset is recorded and ocular artifacts are removed beforehand, canonical microstate topographies and features on eyes open / eyes closed resting state data are robust to muscle-related and non-physiological artifacts and can automatically capture brain-related physiological data.

## Data Availability

No datasets were generated or analysed during the current study.
